# The Effects of Additional Filtration on Image Quality and Radiation Dose in Cone Beam CT: An In Vivo Preliminary Investigation

**DOI:** 10.1155/2022/7031269

**Published:** 2022-03-02

**Authors:** Jan Houfrar, Bjorn Ludwig, Dirk Bister, Manuel Nienkemper, Ciamak Abkai, Adith Venugopal

**Affiliations:** ^1^Department of Orthodontics, Saarland University, Homburg/Saar, Germany; ^2^Department of Orthodontics, Guy's and St Thomas' NHS Foundation Trust and King's College Dental Institute, London, UK; ^3^Department of Orthodontics, Heinrich-Heine-University, Düsseldorf, Germany; ^4^Private Practice, Traben-Trarbach, Germany; ^5^Department of Orthodontics, Saveetha Dental College, Saveetha Institute of Medical and Technical Sciences, Saveetha University, Chennai, India; ^6^Department of Orthodontics, University of Puthisastra, Phnom Penh, Cambodia

## Abstract

**Purpose:**

The aim of this study was to investigate the effect of reduced radiation doses on the image quality of cone-beam computed tomography scans and the suitability of such imaging for orthodontics, oral surgery, dental implantology, periodontology, and endodontology.

**Materials and Methods:**

Cone-beam computed tomography scans of a live patient were performed using seven attenuation filters with increased thickness to decrease the effective radiation dose from 22.4 to 1.8 *μ*Sv, and the effects of different radiation doses on image quality were further analysed. Quantitative image quality was calculated using dedicated measures, such as signal and contrast-to-noise ratio and sharpness. A panel of five certified raters assessed the cone-beam computed tomography scans qualitatively. Nine anatomical structures relevant to dentistry were identified, and the overall acceptance was assessed.

**Results:**

Linear reduction of the effective radiation dose had a nonlinear effect on image quality. A 5-fold reduction in the effective dose led to acceptable quantitative and qualitative image quality measures, and the identification rate of dental anatomical structures was 80% or greater. The use of less than 40% of the reference dose was unacceptable for all dental specialties.

**Conclusions:**

The ideal radiation dose for specific diagnostic requirements remains a patient-related and specialty-related decision that must be made on an individual basis. Based on the results of this study, it is possible to reduce exposure in selected patients, and at the same time obtain sufficient quality of images for clinical purposes.

## 1. Introduction

Cone-beam computed tomography (CBCT) was introduced in 1998 [1] and has since been used in all dental disciplines [2, 3], and specific indications have been identified in orthodontics [4], oral surgery [5], dental implantology [6], periodontology [7, 8], and endodontics [9, 10]. CBCT provides three-dimensional (3D) images, which are represented two-dimensionally, and can add valuable diagnostic information [11, 12]; however, the effective radiation dose increases with image quality [13], and clinicians are advised to use ionizing radiation with the lowest achievable radiation dose for safety purposes [14].

During CBCT scans, patients are exposed to radiation doses between 11 and 374 microsievert (*μ*Sv) [15, 16] that are significantly higher than those in dental panoramic tomography (DPT) or other routinely used imaging modalities in the maxillofacial area (5–15 *μ*Sv) [17–19]. The applied dose depends on the physical process of radiation production, the irradiated area, and the sensitivity of the radiation-detecting equipment. However, the diagnostic value of an image not only is determined by the radiation dose but also depends on the equipment on which the image is visualized, as well as the person who assesses the image [20–23].

Physical parameters, such as beam quality and dose, determine the image quality of radiographs [14, 24–28]. Some image quality parameters, such as signal-to-noise ratio (SNR), contrast-to-noise ratio (CNR), and sharpness, can be objectively measured; thus, subjective image quality is of essential importance and may have a critical impact on diagnosis and treatment planning. A number of studies have often used dry skull phantoms to determine subjective image quality [20, 21, 23, 24]. However, images obtained from dry skulls differ considerably from live patient images because of the absence of soft tissues; therefore, a dry skull model is poorly appropriate for clinical settings [24]. Hence, this study aimed to acquire CBCT images from a live patient using interchangeable filters and to reduce the effective radiation dose. In addition, we also attempted to determine the effect of radiation doses on subjective image quality and assess the ability of such imaging to identify anatomical structures. The images used for this study were acquired with a commercially available CBCT machine that had been modified using seven copper filters.

## 2. Methods

### 2.1. Ethical Approval

The study was performed on a live individual who is one of the authors of this study. He had a skiing accident that resulted in a fractured #21 and warrented a CBCT scan as a part of his clinical care. The subject was assessed by a psychiatrist and found to be competent to evaluate the risks and benefits and to accept full responsibility for the conduct of the experiment. The Declaration of Helsinki does not comment on self-experimentation. The requirement for ethics approval therefore does not apply. Nevertheless, approval for the series of radiographs was obtained from the Trier District Dental Association Public corporation Loebstrasse 18, 54292 Trier. Since the author was also the subject, the requirement for informed consent does not apply. But for the purpose of publication of data, the patient provided an explicit informed consent to participate in the study.

### 2.2. Imaging

A fully dentate live patient was included in this study. No artefacts due to metal objects were visible on CBCT images.

### 2.3. CBCT Unit Preparation and Acquisition of Data Sets

Orthophos® XG 3D (Sirona, Bensheim, Germany) was used for imaging. For dose reduction, a series of seven copper (Cu) filters (F1-F7) (10 mm × 10 mm in size) with different thicknesses (Table 1) were used to attenuate the radiation beam; they were mounted as close as possible to the radiation source. The effective radiation dose for F0 (no filter) was 36 *μ*Sv (Ludlow et al. [29]) and was used to calculate the interpolated effective dose values by linear regression, which was performed based on air kinetic energy released per unit mass measured using a PTW Nomex® ionization chamber (PTW, Freiburg, Germany) in the central line of the beam at the detector's iso-center. The same instrument parameters (7 mA and 85 kV) were used for all imaging experiments.

The field of view was 8 cm × 8 cm with a voxel size of 0.160 mm^3^. Eight different 3D data sets were obtained and stored in Digital Imaging and Communications in Medicine (DICOM) format (one for each filter setting) [30]. All the scans were done in under 10 minutes without changing the setups for each scan. Furthermore, the minor head movements were controlled with the support of the headrest.

### 2.4. Materials Used for Rating

DICOM data was used throughout the study without any image modification/processing. To standardize the images for the ratings, three image sections (A, coronal view section of the lower first molar; B, mandibular axial; and C, maxillary axial) were prepared from each volume dataset. These sections were chosen because they represented the same anatomical location and orientation, as demonstrated in other investigations [20]. The three specific sections depict relevant anatomy for different dental specialties considered in our study. A total of 24 slices were used.

**Figure 1:**
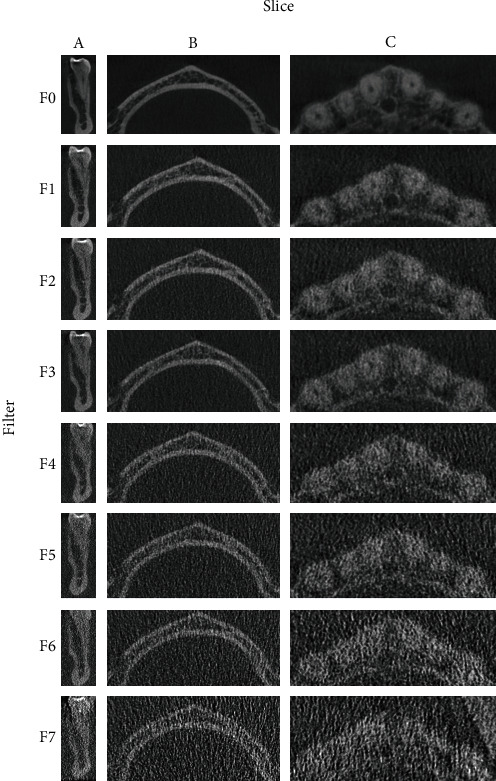
Overview of the 3 slices from 3D CBCT using filters F0-F7. Slice A, coronal view of the lower first molar; slice B, mandibular axial; slice C, maxillary axial.

An overview of the 24 slices with relevant filtration settings (F0-F7) for the patient is presented in Figure 1. Images were used without enhancement to achieve a standardized rating environment. Three slices (A, B, and C) were arranged next to each other for every filtration setting. The slides of different attenuations were randomly distributed for blind assessment to prevent preconditioning during the evaluation phase. The contrast and brightness settings were kept constant.

### 2.5. Qualitative Evaluation

Five CBCT-certified senior dentists at the Dental University Hospital undertook a qualitative analysis of images. The assessors were given verbal and written instructions on how to view and assess/rate the images using custom questionnaires. The images were presented in a randomized order, no time limit was set, and a calibrated and certified diagnostic monitor (terra® LCD 2430W, Wortmann AG, Hüllhorst, Germany) was used under standardized conditions.

Based on the questionnaire, subjective image quality was scored using a five-point rating scale (Q1-Q5) (Liang et al. [20]): 1 = excellent, 2 = good, 3 = acceptable, 4 = poor, and 5 = very poor. Evaluators were asked to identify the following nine dental and anatomical structures (A1-A9): A1, mental foramen; A2, mandibular canal; A3, cortical bone; A4, dental pulp; A5, dentin, A6, incisal canal; A7, enamel; A8, periodontal ligament; and A9, cancellous bone. Identification of anatomical structures A1-A9 was scored as “yes” or “no.” All examiners were asked to assess whether these images were appropriate for the following specialties: S1, orthodontics; S2, oral surgery; S3, dental implantology; S4, periodontology; and S5, endodontology.

### 2.6. Objective Image Quality

**Figure 2:**
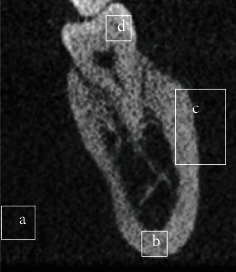
Example of areas used for quantitative image analysis. (a) Background noise; (b) signal bone; (c) signal dentine; (d) the bone soft-tissue edge for sharpness analysis.

Objective image quality was analysed based on three tissues (bone, dentin, and soft tissue) on the anatomical sections (Figure 2). The following three key metrics were analysed.

(1) *SNR*. The SNR was calculated as shown below (equation (1)). The mean value of the signal (*μ*_signal_) was measured from the bone, and the standard deviation of the background noise (*σ*_background_) was calculated from the soft tissue
  (1) $$\begin{array}{c} \text{SNR}=\frac{\mu_{\text{signal}}}{\sigma_{\text{background}}}. \end{array}$$
(2) *Contrast-to-Noise Ratio (CNR)*. The CNR was calculated as shown in equation (2). The mean signal values were measured for the dentine (*μ*_*A*_) and bone (*μ*_*B*_); the background noise (*σ*_background_) was measured from the soft tissue
  (2) $$\begin{array}{c} \text{CNR}=\frac{\;|\;\mu_{A}-\mu_{B}\;|\;}{\sigma_{\text{background}}}. \end{array}$$
(3) The sharpness and edge visibility were calculated as shown in equation (3) on a hard bone-soft tissue edge based on the variation of the 2D line spread function, which is expressed by the 2D gradient of the image (∇*f*(*x*))
  (3) $$\begin{array}{c} \text{sharpness}=\frac{\sigma \left(\;|\;\nabla f\left(x\right)\;|\;\right)}{\sigma_{\text{background}}}. \end{array}$$

### 2.7. Statistical Analysis

Intra- and interexaminer reliabilities were calculated using the intraclass correlation coefficient based on two separate measurements taken four weeks apart: they were 0.80 and 0.77, respectively. Nonlinear regression was used to calculate the association between the mean detection rate and subjective image quality. Qualitative measures from observer ratings are presented as means and standard deviations. Statistical analyses were performed using SPSS® for Windows, version 22.0 (IBM Corp., Armonk, New York, USA). Statistical significance was set at *p* < 0.05.

## 3. Results

**Figure 3:**
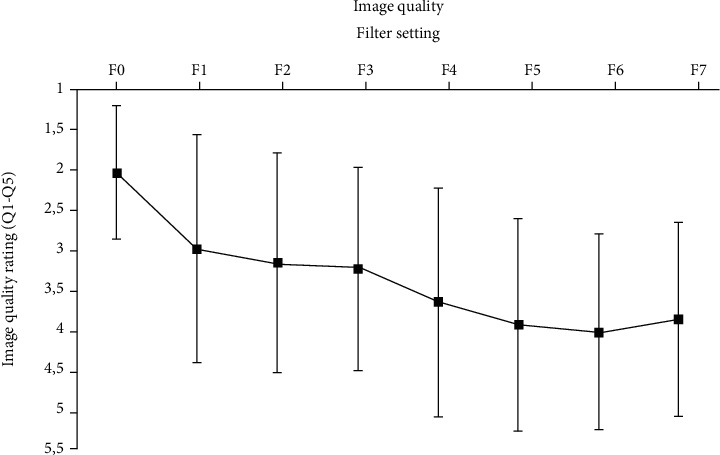
Image quality ratings for all anatomical structures (Q1 to Q5).

A detailed data analysis of image quality for anatomical structures is shown in Table 2. The cortical bone (A3) and the incisal canal (A6) had the best image quality, whereas the periodontal ligament (A8) had generally poor image quality.  Figure 3 shows the mean image quality ratings for all anatomical structures.

**Figure 4:**
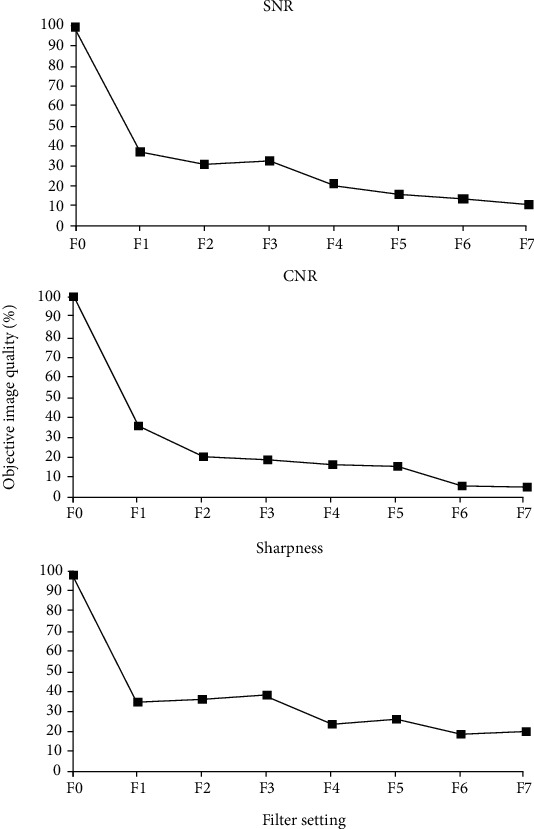
Overall subjective image quality ratings. All assessors' quality ratings (Q1-Q5) for the anatomical structures (A1-A9) investigated are presented as means and standard deviations.

**Figure 5:**
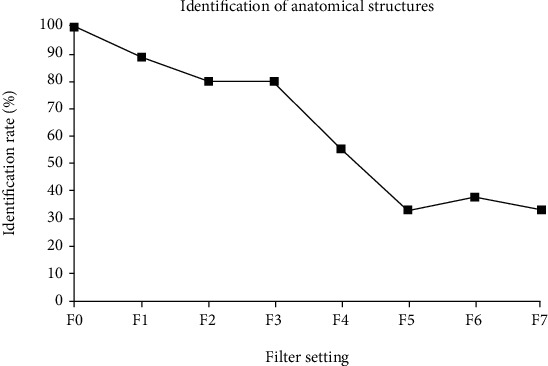
Identification of anatomy. The identification rates of anatomy and all anatomical structures (A1-A9) are shown.

Figure 4 shows objective image quality. Image noise was more pronounced for filters higher than F4; SNR and CNR markedly decreased for doses less than 10% of the reference dose. However, it became difficult to measure sharpness when image noise was high, so differentiation between filters F4-F7 was not performed. For filter settings F0-F3, the identification rates were between 80% and 100% (Figure 5).

The perceived usefulness for different dental specialties (S1-S5) is shown in Table 3. CBCT data acquired in this study were most suitable for orthodontics (S1) but were least suitable for endodontics (S5). Filter settings F3-F7 were rated as inappropriate for all specialties.

**Figure 6:**
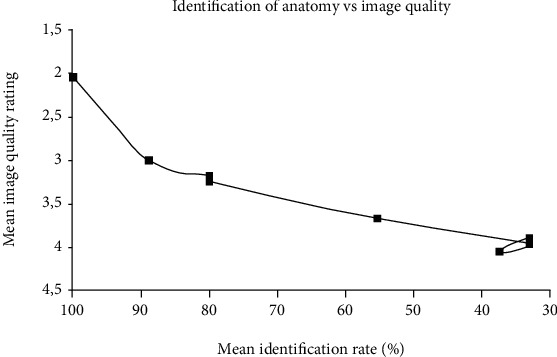
Correlation between the identification rate of anatomy and subjective image quality (F0-F7).

The relationship between the identification rate of anatomy and image quality (A1-A9) is shown in Figure 6. Notably, the plots followed a polynomial function of the second order; in other words, the relationship was not linear.

## 4. Discussion

This investigation assessed the subjective image quality of attenuated CBCT images and their ability to identify anatomical structures. We attempted to determine if CBCT radiographs with reduced effective radiation doses were still able yet maintain the image quality and identify anatomical structures. To the best of our knowledge, this study is the first to analyse the relationship between the identification rate of anatomy and image quality of the attenuated CBCT on a live dentate patient.

For the purpose of this study, we had specifically chosen regions that represented the most relevant structures in different dental disciplines, for example, cortical bone, cancellous bone, root, crowns, enamel, foramina, and other structures of diagnostic interest.

The patient received a combined effective radiation dose of 96 *μ*Sv for all images (F0-F7, exposures range from 1.8 to 36 *μ*Sv based on interpolation). The radiation burden to the patient in this study was lower than that of other commercially available equipment that could expose the patient to 674 *μ*Sv [15]. The copper filters F1-F3 used in our investigation led to effective radiation doses of 22.4, 13.8, and 9.4 *μ*Sv, respectively. Notably, the effective radiation dose for a digital full-size DPT and the cephalometric view is approximately 15 *μ*Sv [17, 19], and the effective radiation dose of an intraoral radiograph is approximately 5 *μ*Sv [18].

### 4.1. Image Quality and Identification of Anatomy

In our investigation, subjective and objective image quality measures decreased with increased thickness of the copper filters (i.e., reduced radiation dose); the findings further confirm that an increase in the radiation dose improves the image quality [13]. However, ratings on subjective image quality showed considerable variations (Figure 3). These variations may have been due to the random order of presentation of the images. The mean identification rate of anatomy (Figure 5) for filter settings F1-F3 was high. The raters were able to identify 80% of anatomy structures (A1-A9). Objective image quality (SNR, CNR, and sharpness) and subjective image quality were similar for filters F1-F3 (Figure 4). The modulation transfer function (MTF) was not used because it has been shown to be less robust for background noise [31]. These results highlight that the use of filters F1-F3, which correspond to 62%, 38%, and 26% of the reference dose, respectively, did not lead to a linear decrease in image quality; the image quality was consistent with all criteria (SNR, CNR, sharpness, subjective image quality, and mean identification). However, the use of filters F4-F7 led to a remarkable decrease in image quality, indicating a limited clinical availability of filters F4-F7. Notably, additional scattering effects caused by the filters were unlikely to influence our present results since the copper plates were very thin [32].

### 4.2. Applicability in Dental Specialties

Accumulating evidence indicates that CBCT is a very valuable imaging modality for endodontics and has been considered to have a significant impact on diagnosis and treatment planning. However, CBCT is currently only recommended for a small group of patients with complex endodontic problems [9]. Differences between different anatomical sites (mandibular vs. maxillary, anterior vs. posterior, etc.) needs related to the patient, and the specific clinical situation may have different implications on the outcome and the usefulness of the images in dentistry.

In our study, the assessors found that CBCT images obtained from filters F1-F3 were best suited for orthodontics, oral surgery, and dental implantology, in a descending order. Since periodontology, and endodontics dealt with very small anatomical structures, the images from the filters were deemed inappropriate. However, the assessors rated the data sets obtained from filters F3-F7 as inappropriate for any of the dental specialties (S1-S5) considered in our study. This finding suggests that the attenuated CBCT imaging technique is unlikely to be clinically used in dental specialties relying on the identification of small (micro) structures, such as the periodontal ligament and the root canal system.

### 4.3. Correlation of the Identification Rate of Anatomy with Image Quality

The nonlinear regression showed a good correlation between identification of anatomy and image quality (*R*^2^ = 0.92, *p* < 0.001). These findings demonstrate the usefulness of the attenuated CBCT imaging technique, despite the relatively small number of evaluators. Despite the overall poor median image quality measures, F0 settings exhibited good identification, except for small anatomical structures A4 (dental pulp) and A8 (periodontal ligament). The usefulness of the images for endodontics (S5) and periodontology (S4) was rated lowest.

### 4.4. Strengths and Limitations of the Study

The CBCT technique in this study was designed to standardize the testing conditions. However, the slides presented to the evaluators were not necessarily relevant to the dental subspecialties. Indeed, scrolling through all the CBCT images leads to a better representation of 3-dimensional structures on a 2-dimensional monitor and allows for changes in brightness, contrast, and different settings of the Hounsfield units (HU).

Although the assessors were certified CBCT image raters, our present results only reflect their subjective impressions of image availability for different dental specialties. Whether an image is considered acceptable for clinical use often depends on the subjective evaluation of clinicians [20–23], regardless of whether an image can be modified for viewing. Our findings confirm that the reduced CBCT radiation dose may still allow reliable assessment of the anatomy [23, 33, 34] as long as it is well indicated for a particular specialty.

The assessors considered 25% of CBCT images as inappropriate. The findings are not consistent with those in some current studies, which suggest that CBCT-derived cephalometry is generally comparable, despite not being more reliable [35–41]. Our investigation did not determine the accuracy of amalgamated CBCT images compared with conventional cephalometry. We assessed noncephalometric landmarks on CBCT images using modified equipment with reduced radiation burden. With the equipment used in our study, the radiation burden of CBCT was roughly equivalent to that of combined conventional DPT and cephalometric radiograph [17–19]. This finding suggests that the combined conventional DPT and cephalometric radiograph, which have often been used as part of the initial standard diagnostics [42], could be replaced by CBCT. However, this assumption is only correct if all images are of diagnostic quality. Further studies are needed to determine the reliability of CBCT images acquired by the modified equipment used in our study. Furthermore, different types of filters may have varying effects on the reduction of the subject's radiation exposure as well as the quality of the images obtained. This could be a possible evaluation for further studies. Using filters F1-F3, which were comparable to 62–26 percent of the reference dose, was possible and resulted in reliable anatomical structure identification. As a result, a further dose reduction within this range could be intriguing for future research.

## 5. Conclusion

Higher radiation doses led to better objective and subjective image quality and identification ratings. However, the relationship between the applied radiation dose and image quality measures was nonlinear. In addition, the use of filters F1-F3, which were equivalent to 62–26% of the reference dose, was feasible and still resulted in reliable identification of anatomical structures. However, image quality decreased markedly for filter settings where less than 11% of the reference dose was used. Moreover, attenuated CBCT was considered acceptable for orthodontics, oral surgery, and dental implantology, but not for periodontology and endodontics. While the loss of image quality may be acceptable for some indications, such imaging approaches cannot be recommended for imaging small anatomical structures, such as the periodontal ligament and the root canal system. Our findings suggest that the best radiation dose for specific diagnostic requirements remains a specialist-patient related decision, which has to be made on an individual basis, and it is possible to reduce exposure in selected patients, and at the same time obtain sufficient quality of images for clinical purposes.

## Figures and Tables

**Table 1:** Filter settings and effective and relative radiation doses.

Filters	Cu-filter thickness (mm)	Air kerma (*μ*Gy)	Relative absorbed dose (%)	Interpolated effective dose∗ (*μ*Sv)
F0	0 (no filter/reference)	1255	100	36†
F1	0.2	779	62	22.4
F2	0.4	480	38	13.8
F3	0.7	328	26	9.4
F4	1.0	202	16	5.8
F5	1.3	134	11	3.8
F6	1.5	104	8	3.0
F7	2.0	63	5	1.8

F0 (no filter) was used as the reference dose. Relative absorbed doses were assessed based on ion chamber dosimetric (air kerma) measurements with a repeatability error of <0.03-1%. Interpolated effective doses were calculated as linear interpolation in relation to F0. ^†^Taken from Ludlow et al. [29]. ^∗^Interpolation was calculated based on relative absorbed dose measurements and a real effective dose reference value.

**Table 2:** Image quality rating of anatomical structures A1-A9 for filter settings F0-F7.

	F0	F1	F2	F3	F4	F5	F6	F7
Anatomical structures	M ± SD	M ± SD	M ± SD	M ± SD	M ± SD	M ± SD	M ± SD	M ± SD
A1	1.4 ± 0.9	3.2 ± 2.0	3.0 ± 1.6	2.8 ± 1.8	3.6 ± 1.7	4.2 ± 1.8	3.0 ± 2.0	3.4 ± 2.2
A2	2.8 ± 1.3	2.6 ± 1.1	3.2 ± 1.3	4.0 ± 1.2	4.6 ± 0.9	4.6 ± 0.5	4.8 ± 0.4	4.8 ± 0.4
A3	1.6 ± 0.5	2.2 ± 1.8	2.6 ± 1.1	2.8 ± 1.6	3.0 ± 1.6	3.2 ± 2.0	3.4 ± 1.8	3.2 ± 1.8
A4	3.0 ± 1.0	3.4 ± 1.3	3.6 ± 1.3	3.0 ± 1.0	4.0 ± 1.4	4.0 ± 1.2	4.4 ± 0.9	4.0 ± 1.0
A5	2.0 ± 0.7	3.4 ± 1.3	3.4 ± 1.5	3.4 ± 1.1	3.4 ± 1.5	3.8 ± 1.3	4.4 ± 0.9	4.2 ± 0.8
A6	1.4 ± 0.5	1.8 ± 0.8	2.8 ± 1.3	2.6 ± 1.1	3.4 ± 1.5	3.2 ± 2.0	3.6 ± 1.9	3.4 ± 1.7
A7	1.8 ± 0.8	3.0 ± 1.4	3.0 ± 1.6	3.2 ± 1.3	3.0 ± 1.6	3.8 ± 1.3	4.2 ± 1.1	3.8 ± 1.1
A8	3.0 ± 1.2	3.8 ± 1.6	3.6 ± 1.3	4.0 ± 1.0	4.2 ± 1.1	4.4 ± 0.9	4.4 ± 0.9	4.2 ± 0.8
A9	1.4 ± 0.5	3.6 ± 1.3	3.4 ± 1.3	3.4 ± 1.1	3.8 ± 1.6	4.4 ± 0.9	4.2 ± 1.1	4.0 ± 1.0

A1: foramen mentale, A2: mandibular canal, A3: cortical bone, A4: dental pulp, A5: dentine, A6: incisal canal, A7: enamel, A8: periodontal ligament, and A9: cancellous bone. All assessors' (*n* = 5) quality ratings are presented as means (M) and standard deviations (SD).

**Table 3:** Perceived usefulness ratings.

Perceived usefulness ratings (%)								
	F0	F1	F2	F3	F4	F5	F6	F7
S1	100	40	40	0	0	0	0	0
S2	100	40	20	0	0	0	0	0
S3	80	20	40	0	0	0	0	0
S4	60	0	40	0	0	0	0	0
S5	60	0	0	0	0	0	0	0

Perceived usefulness ratings (%) and different dental specialties depended on filter settings F0-F7. S1: orthodontics; S2: oral surgery; S3: dental implantology; S4: periodontology; S5: endodontics.

## Data Availability

The data used to support the findings of this study are available from the corresponding author upon request.
